# Comparing accuracy in guided endodontics: dynamic real-time navigation, static guides, and manual approaches for access cavity preparation – an in vitro study using 3D printed teeth

**DOI:** 10.1007/s00784-024-05603-8

**Published:** 2024-03-14

**Authors:** Karin Christine Huth, Lukas Borkowski, Anja Liebermann, Frank Berlinghoff, Reinhard Hickel, Falk Schwendicke, Marcel Reymus

**Affiliations:** 1 Department of Conservative Dentistry and Periodontology, University Hospital, LMU Munich, Goethestr. 70, 80336 Munich, Germany; 2 Department of Prosthetic Dentistry, Faculty of Medicine, University of Cologne, University Hospital Cologne, Kerpener Str. 32, 50931 Cologne, Germany

**Keywords:** Access cavity preparation, Dynamic navigation, Guided endodontics, 3D printing, Static navigation, Surgical guide, Template

## Abstract

**Objectives:**

To assess root canal localization accuracy using a dynamic approach, surgical guides and freehand technique in vitro.

**Materials and methods:**

Access cavities were prepared for 4 different 3D printed tooth types by 4 operators (*n* = 144). Deviations from the planning in angle and bur positioning were compared and operating time as well as tooth substance loss were evaluated (Kruskal-Wallis Test, ANOVA). Operating method, tooth type, and operator effects were analyzed (partial eta-squared statistic).

**Results:**

Angle deviation varied significantly between the operating methods (*p* < .0001): freehand (9.53 ± 6.36°), dynamic (2.82 ± 1.8°) and static navigation (1.12 ± 0.85°). The highest effect size was calculated for operating method (ηP²=0.524), followed by tooth type (0.364), and operator (0.08). Regarding deviation of bur base and tip localization no significant difference was found between the methods. Operating method mainly influenced both parameters (ηP²=0.471, 0.379) with minor effects of tooth type (0.157) and operator. Freehand technique caused most substance loss (*p* < .001), dynamic navigation least (*p* < .0001). Operating time was the shortest for freehand followed by static and dynamic navigation.

**Conclusions:**

Guided endodontic access may aid in precise root canal localization and save tooth structure.

**Clinical relevance:**

Although guided endodontic access preparation may require more time compared to the freehand technique, the guided navigation is more accurate and saves tooth structure.

**Supplementary Information:**

The online version contains supplementary material available at 10.1007/s00784-024-05603-8.

## Introduction

Tooth structure conserving endodontic access cavity preparation and accurate root canal localization are the foundation for a successful root canal treatment. Challenges arise in complex cases, like finding obliterated second mesio-buccal root canals in upper molars, additional canals in mandibular canines or dealing with calcifications and pulp stones [1–5]. Treating such cases is time-consuming and often go hand in hand with higher substance loss and risk of perforation or missed canals. This leads to endodontic failure and reduced fracture resistance [6–11]. To address these challenges, static and dynamic guided endodontic systems utilizing treatment planning based on cone beam computed tomography (CBCT) contribute to precise root canal orifice localization [12–18]. In the software, the bur’s virtual placement at the orifice ensures direct access, minimizing substance loss even without direct sight. Static navigation has been explored in numerous prior studies, primarily consisting of case reports or in vitro investigations utilizing either human or 3D-printed teeth [19–21]. For this method the CBCT-based planning is merged with a surface scan, and a template for guided drilling is fabricated, either by subtractive or additive manufacturing. On the contrary, dynamic navigation is a relatively new area of modern digital dentistry, yet it is increasingly apparent in a vast spectrum of dental procedures [22], including endodontic treatments for obliterated root canals [23] or endodontic surgery [24]. It integrates a visual marker during CBCT and operation, bypassing the need for surface scans. The marker is detected by an external camera, serving as a reference point for overlaying CBCT data, guiding real-time drill positioning as per the plan [25, 26]. Dynamic navigation’s key advantage lies in intraoperative adjustments, in contrast to static guides, which may be required due to CBCT misinterpretation [27]. Without physical templates, the procedure area remains visible, proper cooling is maintained, cavity rinse and rubber dam placement is possible, same-day treatment is viable, and even changes in planning are feasible [16, 23]. A potential downside could be a steeper learning curve, as operators juggle display viewing and on-site drilling challenges [26, 28–30]. Static or dynamic guidance’s advantages are evident in localizing calcified root canals [12, 30–34]. However, extending the research to the use of guided endodontics in complex root canal anatomies, such as clinicians are confronted with during treatment, is the aim of this study, thereby using 3D printed teeth.

This study compared the accuracy of CBCT-based dynamic and static navigation systems, and the freehand technique for access cavity preparation in anatomically challenging 3D printed replica teeth. The null hypothesis was that there is no difference in accuracy regarding CBCT-based planning and actual access cavity preparation among the three methods.

## Materials and methods

**Fig. 1:**
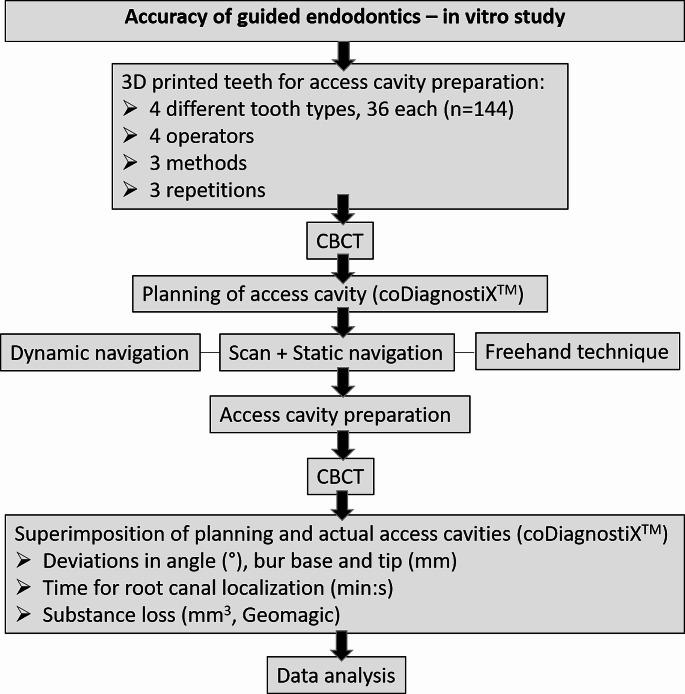
Study design

The study was conducted at the Department of Conservative Dentistry and Periodontology, University Hospital, LMU Munich, from 2020 to 2022, and approved by the local ethics committee (No 21–0820) for the use of human teeth. The study design is depicted in Fig. 1.

3D printed replica teeth were crafted from CBCT data (CBCT Carestream CS9300, Carestream Dental, Atlanta, USA; voxel size 0.090 mm) of 4 extracted human teeth, each with a specific complex anatomy. The selected replica presented procedural challenges that dentists face in daily practice, and originated from a hodgepodge of training teeth for undergraduate and postgraduate students. (1) Upper right canine with one root canal obliterated till the middle apical third. (2) Upper second left molar with an obliterated second mesio-buccal root canal to be localized (3) Lower left molar with a pulp chamber containing pulp stones with the distal root canal to be found. (4) Lower right canine having a second lingual canal which needed access.

The CBCT images were segmented (3D Slicer software) and corresponding DICOM data converted into STL files [35, 36]. Cubes around the roots were added (Meshmixer software, Autodesk, San Rafael, CA, USA) for facilitating later precise model alignment. Each tooth was reproduced 36 times using a stereolithographic printer (rapidshape D20+, Rapid Shape GmbH, Heimsheim, Germany). A special resin was used to ensure sufficient radio-opacity of the replica teeth (V-Print, VOCO GmbH, Cuxhaven, Germany). One of each replica tooth type was accurately positioned within a wax jaw model (Nissin Dental Products INC., Kyoto, Japan). After removing the 3D printed teeth the model was duplicated using polysiloxane and duplicated using high-precision model stump material (Picopoly, picodent, Wipperfürth, Germany). Subsequently, the replica teeth were reinserted into the final models. The printed replica teeth were randomly assigned to 3 groups with 12 teeth per type each: (a) Dynamic navigation group using the Denacam system (mininavident AG, Liestal, Switzerland); (b) Static navigation group using a printed static surgical guide (coDiagnostiX, Dental Wings INC, Montreal, Canada); (c) Freehand technique group.

For the dynamic navigation group, a pre-op CBCT was taken for each model (Carestream CS9300) with a ceramic marker [26]. Data was exported as DICOM-file. For Group SAC, an optical scan (Activity 885 Mark 2, Pluradent GmbH. & Co. KG, Offenbach, Germany; .stl dataset) was conducted and aligned using spheric landmarks. For access planning, a CBCT scan of the model was uploaded to a planning software (coDiagnostiX™, Dental Wings INC., Montreal, Canada). The respective canal was superimposed with a virtual endodontic bur (Spiralbur Endo, Ref.: O.27.28.B044.051, Steco-system-technik GmbH & Co. KG, Hamburg, Germany; diameter 1 mm, working length 21 mm) ending shortly coronal from the root canal and integrating a sleeve for SAC (Sleeve Guided Endo Ref.: M.27.28.D100L5). For better comparison the same bur was used for the dynamic approach. Group DAC data was exported as .genexa files to Denacam. For the static navigation group, guides were designed in coDiagnostiX™, then 3D printed (V-Print SG, VOCO GmbH, Cuxhaven, Germany). The freehand technique group involved digital planning only.

Four dentists using 3-fold magnifying loops performed the access preparations with the models fixed to a phantom head (Frasaco, Tettnang, Germany). All operators possessed over 5 years of experience in general dentistry including endodontics with freehand access preparation. While they had occasionally utilized static navigation before, they lacked experience with dynamic navigation. To address this, operators underwent calibration via a theoretical and practical tutorial on Denacam and drilling templates provided by the respective manufacturer. Each operator conducted five access preparations on similar teeth during the calibration process. Within the study itself, each dentist performed three repetitions of each operating method with a two-week interval, treating a total of 36 replica teeth (4 teeth x 3 operating methods x 3 repetitions). The CBCT images and measurements were performed by a single operator who held the necessary license for capturing CBCT images and was proficient in operating the CBCT device. Furthermore, this operator received theoretical and practical training on conducting CBCT based measurements from the manufacturer of the coDiagnostiX™ software. Before the study commenced, the operator processed measurements for five separate teeth.

The Denacam system application has been outlined before [15, 26]. After registering the endodontic bur, the model received a marker. On-screen views displayed CBCT-based sagittal/horizontal sections, and target graphic of the planning. Drilling with a green contra-angle handpiece revealed real-time deviation from planning (entry point, angle, depth).

In the static navigation group, the surgical guide was positioned, and the bur was introduced with 5,000 rpm in picking movements until contact with the template’s metal sleeve.

In the freehand technique group, operators received the CBCT scan for planning before treatment.

For each operating method root canal localization was controlled by instrumenting the root canal with a size 10 K-file (VDW).

**Fig. 2:**
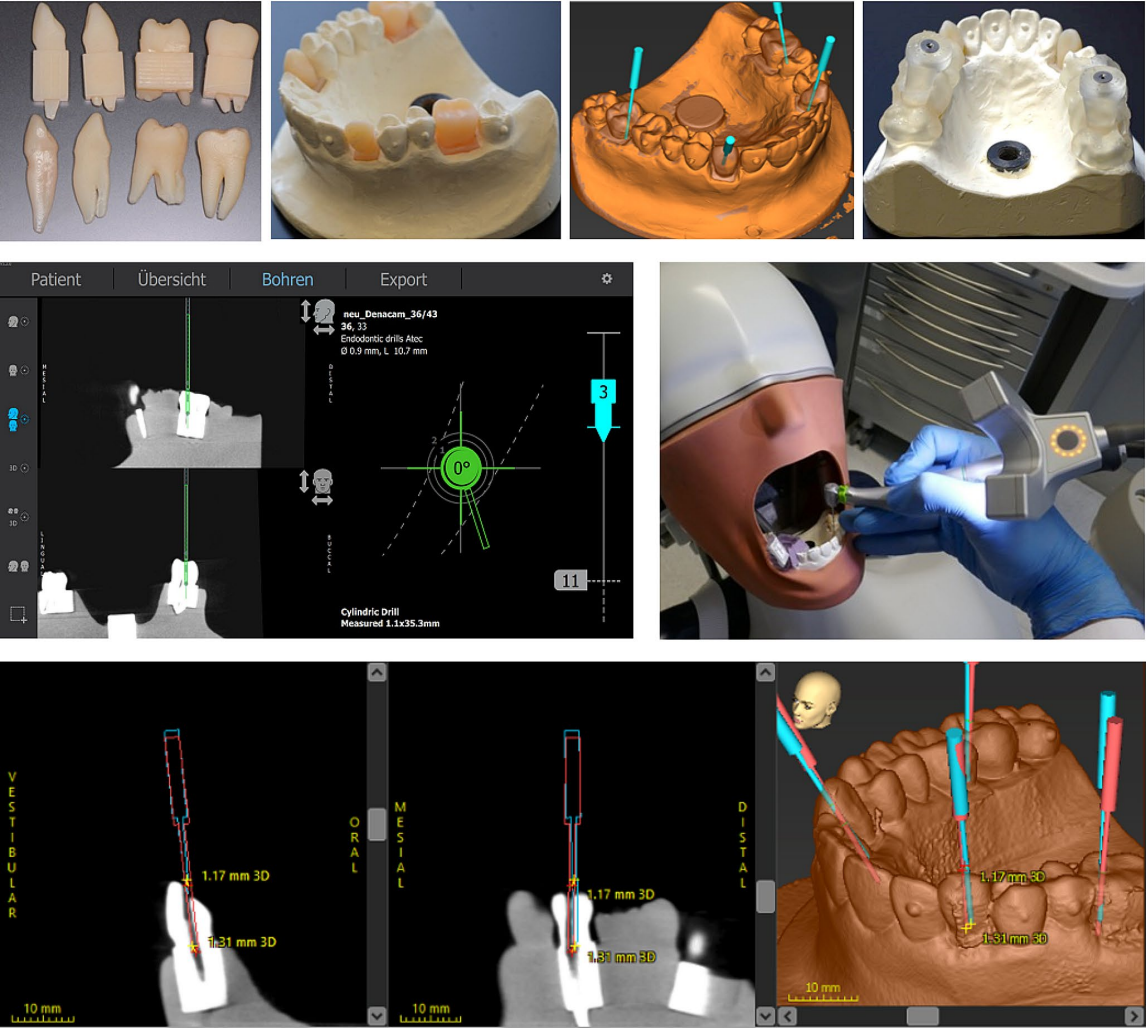
Upper row (left to right): 3D printed teeth with and without cubes around the roots; model with integrated replica teeth; planning of access cavity for the replica teeth (coDiagnostiX™); 3D-printed guides for static navigation positioned on the model. Middle row (left to right): Screen view providing all information during dynamic navigation of access cavity preparation (CBCT-based horizontal/sagittal section on the left side and target graphic of the planning regarding angle and drill depth on the right side); dynamically navigated drilling of the access cavity at the phantom head using the Denacam system. Lower row: Superimposition of the planned (blue) and actual (red) drill path within the coDiagnostiX™ software. The 3D deviations at the bur’s base and tip (mm) are shown

After cavity preparation, another CBCT scan was conducted, imported into coDiagnostiX™ and aligned with preoperative plannings, resulting in the output of the following deviations: (1) Bur angle (°), (2) 3D distance at bur entry point (Baseoffset3D) (mm) and (3) apex (Tipoffset3D). The studies procedure from 3D printed teeth to the measurement of the deviations is illustrated in Fig. 2. Additionally, time taken to locate root canal (min:s), and tooth substance loss volume (mm^3^) were evaluated (Geomagic Control 2015 software, 3D Systems GmbH, Mörfelden-Walldorf, Deutschland). Further outcomes were recorded: (1) root canal found (yes/no) and (2) perforation occurrence (yes/no). Inter-examiner reliability was assessed by comparing all operators’ access preparations regarding primary outcome parameters at the study’s end.

Statistics were calculated using SPSS 27.0 (IBM Cooperation). Descriptive statistics was calculated (mean, standard deviation, range, median). Normal distribution was checked using Shapiro-Wilk test. Differences between operating methods were compared by Kruskal-Wallis-Test and One Way ANOVA. Secondary parameters were analyzed with the Chi-square test (alpha level 0.05). A general linear model (partial eta-squared statistic, ηP²) assessed effect sizes of influencing factors (operator, method, tooth type, and interactions). For investigating inter-examiner reliability intraclass correlation coefficient (ICC) was used [37, 38]. For the sample size and power calculation [39], we used the outcome of a previous study [40] regarding angular deviation comparing dynamic navigation, static navigation, and freehand technique as well, resulting in a required sample size of *n* = 47 with the power set at 90%.

## Results

**Fig. 3:**
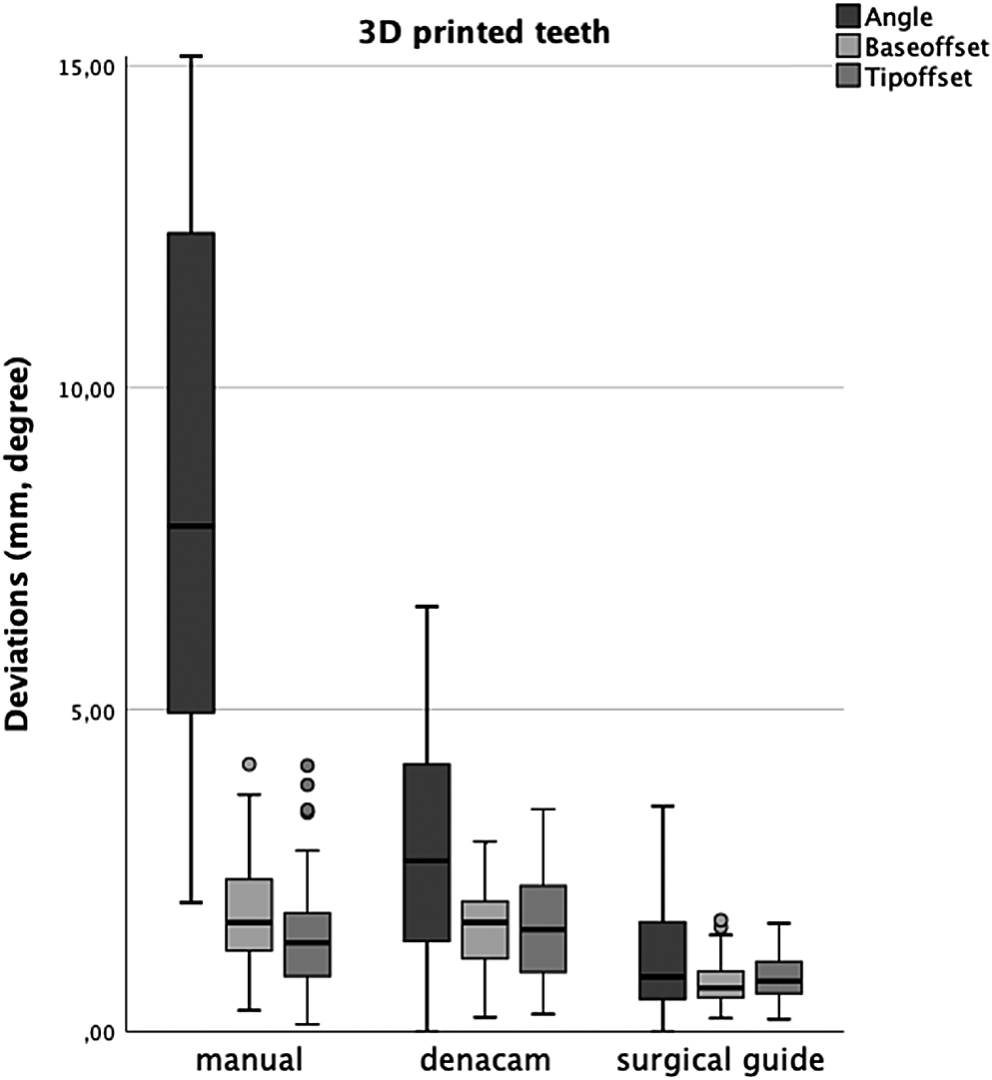
Boxplots of deviations between planned and actual drill paths

Deviations between planned and actual drill positions for each method and secondary outcome results are summarized in Tables 1 and 2 and graphed in Fig. 3.

**Table 1:** Table 1

Parameters	Groups	Mean ± SD	Min	Max	Median	Comparison	*P* value
Angle (°)	Freehand	9.53 ± 6.36	2.00	27.00	7.85	MAC-DAC*	0.000
	Dynamic	2.82 ± 1.8	0.00	6.60	2.65	DAC-SAC*	0.000
	Static	1.12 ± 0.85	0.00	3.50	0.85	SAC*-MAC	0.000
Baseoffset3D	Freehand	1.85 ± 0.89	0.33	4.15	1.69	MAC-DAC	0.476
(mm)	Dynamic	1.62 ± 0.7	0.22	2.95	1.70	DAC-SAC *	0.000
	Static	0.77 ± 0.37	0.21	1.73	0.68	SAC*-MAC	0.000
Tipoffset3D	Freehand	1.52 ± 0.90	0.11	4.13	1.38	MAC-DAC	0.550
(mm)	Dynamic	1.65 ± 0.79	0.27	3.45	1.59	DAC-SAC *	0.000
	Static	0.86 ± 0.38	0.19	1.68	0.78	SAC*-MAC	0.000
Substance loss	Freehand	31.90 ± 19.78	4.96	110.27	31.73	MAC-DAC *	0.000
(mm^3^)	Dynamic	9.43 ± 9.02	0.19	41.84	6.07	DAC *-SAC	0.001
	Static	17.60 ± 10.66	1.56	34.72	14.65	SAC*-MAC	0.001
Time	Freehand	02:17 ± 01:07	00:38	05:10	01:59	MAC*-DAC	0.000
(min:s)	Dynamic	04:12 ± 01:50	01:47	07:19	03:43	DAC-SAC *	0.000
	Static	02:22 ± 00:58	00:51	04:08	02:08	SAC-MAC	0.489

Calculated deviations between planned and actual drill path, tooth substance loss, and the time required to find the root canal. Given are the mean and standard deviation (SD), minimum (min), maximum (max), median, and *p-*value of the comparison between the three methods (freehand technique, dynamic, and static navigation). Methods with significantly better results were identified with (*) indicating significantly less deviation, less substance loss, or less time required

**Table 2:** Table 2

Parameter (n)		3D printed teeth				Success rate, %	
		Yes	No				
Canal found	Freehand	45	3			93.7	
	Dynamic	46	2			95.8	
	Static	47	1			97.9	
Perforation	Freehand	2	46			-	
	Dynamic	1	47			-	
	Static	0	48			-	

Number of root canals found, perforations during drilling as well as total success rates in finding the canal (%)

Angle deviations differed significantly among the three groups (*p* < .0001) (Table 1). The freehand group had the highest deviation at 9.53 ± 6.36° while the dynamic group averaged 2.82 ± 1.8°. The lowest deviation (1.12 ± 0.85°) was seen in the static navigation group. Deviations at the bur base and tip were not significantly different between freehand and dynamic groups (*p* > .05), but static navigation had significantly fewer deviations (*p* < .0001; Table 1). Root canal location time was significantly shorter for freehand technique (02:17 ± 01:07 min) than dynamic (04:12 ± 01:50 min, *p* < .000). Static navigation (2:22 ± 0:58 min) was notably quicker than dynamic (*p* < .000), with no difference between freehand and static (*p* = .489). Despite 6 unfound canals and 3 perforations, no significant inter-group differences (*p* < .05) were observed (Table 2).

In terms of effect sizes, method had the greatest impact on angle deviations between planned and actual drill path (ηP²=0.524), followed by tooth type (0.364), while operator influence was minimal (0.08). Operating method also significantly influenced deviation at the entry point (ηP²=0.471) along with tooth type (0.157), while operator impact was not significant. For the deviation at the bur tip, method (ηP²=0.379) and tooth type (0.21) were the primary influences. Required time was mainly affected by method (ηP²=0.524), followed by tooth type (0.363), and operator (0.203). Interaction terms (method*tooth type, method*operator, operator*tooth type) impacted primary outcomes respectively. Tooth substance loss due to access cavity preparation was highest with freehand (*p* < .001) and least with dynamic navigation (*p* < .000) (Table 1). It was primarily attributed to method (ηP²=0.494), tooth type (ηP²=0.356) and least to operator (ηP²=0.079). Inter-examiner reliability was good among operators: ICC 0.878 (CI: 0.798 − 0.932) for angle deviation, 0.775 (CI: 0.625 − 0.875) for 3D deviation at entry point, 0.796 (CI: 0.663–0.886) for 3D deviation at apex, and 0.798 (CI: 0.657 − 0.889) for time taken. Power calculation for angle deviation differences between methods was 99%.

## Discussion

Guided systems in endodontics are used to access root canals in challenging teeth, aiming to reduce treatment time, risks, and tooth substance loss, which can affect long-term success [4, 13, 41]. This study compared a dynamic navigation system (Denacam), static guides, and the freehand technique for access cavity preparation regarding accuracy (angle, bur base and tip), tooth substance loss and required time. It utilized teeth with challenging anatomy: obliterated second mesio-buccal canal in upper molar, pulp stones in lower molar, second lingual root canal in premolar and obliterated canine.

The null hypothesis, which posited that there was no difference in accuracy among the three methods, must be rejected for most of the comparisons (Table 1). However, the null hypothesis could be accepted regarding the deviations of bur base and tip when comparing the freehand technique and dynamic navigation, as well as regarding the time taken to find the canal when comparing the freehand technique and static navigation.

Regarding static navigation, it was found to offer the most accurate cavity preparation, contrasting with higher angle, entry, and bur tip deviations for the freehand technique. However, the angular difference was mainly prominent due to the lower right canine’s additional root canal. Although computer algorithms suggested a vestibular access, which has been reported before [33, 42], due to several reasons, such as straight-line access and minimal volume loss, the dentist preferred a lingual approach without navigation. This preference likely stems from the reluctance to perforate the vestibular side of the tooth. Nevertheless, this access opening can be easily refilled with today’s highly aesthetic composite filling materials. It appears that dentists still favor traditionally taught access openings despite computer-assisted guidance programs indicating a more effective and safer approach. When comparing our results, using the dynamic navigation (Denacam) with previous studies using X-Guide (X-Nav Technologies) and Navident systems (ClaroNav Inc.) similar trends in angle deviations (2.39 ± 0.85° versus 7.25 ± 4.2° and 2.81 ± 1.53°) and canal findings (success rate 96.6% (29/30); 93%) were revealed [16, 43]. Another recent study using Denacam reported slightly higher canal success rates for both methods (97.2%) [15]. For tooth substance loss, dynamic navigation caused least, followed by static, and freehand technique had the highest, which is in line with earlier findings [15, 44]. Interestingly, similar findings have also been observed in the field of implant positioning. For instance, one study revealed an angle deviation of 3.04° for static navigated implant surgery compared to 7.03° for freehand surgery [45]. Another study found angular deviations of 3.18° for static, 3.28° for dynamic, and 7.5° for freehand implant insertion, respectively [46]. Our study revealed that method had the highest influence on accuracy and substance loss, followed by tooth type; operators’ impact was only minimal. The latter finding can probably be explained by the four operators being rather equally experienced in this study. Interestingly, a recent trial investigated whether operators with different levels of experience performed differently when treating obliterated teeth using a dynamic device rather than the freehand technique and found no difference in success rate [16]. This might strengthen the previously stated hypothesis that the use of dynamic navigation could assist less experienced operators in preserving more tooth structure and achieving better results in terms of time required to locate the canal compared to the freehand technique [15, 16, 18]. However, the present study revealed that the dynamic navigation took the longest, while the freehand technique was the fastest. This observation might be explained by the operators having performed only five test teeth with the dynamic navigation device prior to the study, which is notably less than in a comparable study [16]. While this aspect represents a limitation of our study design, it also corresponds with the reported steeper learning curve and the necessity for training with dynamic navigation devices [16, 44].

Further, dynamic navigation was introduced to address limitations of static templates, such as challenges with rubber dam placement, adequate rinsing of dentin debris, and restricted mouth opening [23, 26, 34, 42, 43]. By offering flexibility and addressing spatial constraints, dynamic navigation has been reported to mitigate these issues, improving outcomes [44–48].

To address challenges in managing dynamic navigation, such as transitioning between viewing displays and on-site drilling, augmented reality is emerging as a solution to overlay virtual planning onto the clinical site using head-mounted devices [22, 49].

While research used to focus mainly on single-rooted obliterated front teeth, and less on sparsely available molars or anatomical issues other than obliterations [18–21, 49], for this study, 3D printed replica teeth with different anatomical challenges were utilized. The advantage of using 3D printed teeth is that they can be replicated on a large scale, allowing for the investigation of specific, and even rare, challenging anatomies or pathological changes [50–52]. On the downside, 3D printing resin is monochromatic, which hampers dentin identification playing a role in finding canal orifices manually, and lacks dentin hardness [36, 50, 51].

Guided endodontics in general shows increasing implications for clinical practice, as has been reviewed in a current expert consensus paper [21], given the rising prevalence of tooth obliterations due to aging, increased use of regenerative endodontic procedures, fiber post insertions/removals, and treatment of teeth with special morphological abnormalities. In terms of clinical implications, our study contributes to the existing knowledge by examining the accuracy and substance loss associated with guided endodontic treatment of teeth with different anatomical challenges using 3D printing. It points to the importance of training when using dynamic navigation to achieve efficiency comparable to the freehand technique. Furthermore, it suggests considering vestibular access even when using the freehand technique to ensure straight-line access in difficult cases.

Given the study’s limitations, including its in vitro design, a limited variety of different teeth, and possibly not enough training of the operators in utilizing the dynamic device, guided endodontics emerges as more accurate with less tooth substance loss compared to freehand cavity preparation, while the freehand technique was fastest. Using 3D printed teeth seems to be valuable for such research. Yet, high-quality clinical studies are needed to validate these findings for both dynamic and static navigation systems.

### Electronic supplementary material

Below is the link to the electronic supplementary material.

Supplementary Material 1

## Data Availability

No datasets were generated or analysed during the current study.
