# Supplementary material for: Comparing accuracy in guided endodontics: dynamic real-time navigation, static guides, and manual approaches for access cavity preparation – an in vitro study using 3D printed teeth
Source: Clin Oral Investig. 2024 Mar 14;28(4):212. doi: 10.1007/s00784-024-05603-8 (PMC10937753; doi:10.1007/s00784-024-05603-8)
Supplement: Supplementary file 1 — Supplementary Material 1 [file 784_2024_5603_MOESM1_ESM.docx]

| Department of Conservative Dentistry and Periodontology, University Hospital, LMU Munich; Goethestr. 70, 80336 Munich, Germany |  |  |  | **Head**  Prof. Dr.med.dent. F. Schwendicke  **Corresponding Author:**  Karin C. Huth  Prof. Dr., MME  Tel. +49 89 4400-59411  Fax +49 89 4400-59302  Karin.Huth@med.uni-muenchen.de |
| --- | --- | --- | --- | --- |
| **Gianrico Spagnuolo**  **Editor**  **Clinical Oral Investigations** |  |  |  |  |
| Manuscript: |  |  |  |  |
| **Comparing Accuracy in Guided Endodontics: Dynamic Real-Time Navigation, Static Guides, and Manual Approaches for Access Cavity Preparation – An In Vitro Study using 3D Printed Teeth** | |  |  |  |

**20th February 2024**

Dear Prof. Spagnuolo,

Thank you for the opportunity to revise our manuscript for possible publication in Clinical Oral Investigations.

We have addressed all raced points within a point-by-point response file to the best of our knowledge.

The manuscript has been approved by all authors. The corresponding author takes full responsibility for the submission and confirms that this work is original, free from any infringement upon copyright or proprietary rights of third parties, not currently under consideration by any other journal, and has not been previously published. We are hopeful that you will find our revised manuscript be of value to your readership, deserving of publication in your esteemed journal.

We have enclosed the revised manuscript with highlighted changes, along with the accompanying tables and figures, and the point-by-point response file. Among the changes are two new figures illustrating the study design and procedure, as requested by reviewer 1. As well, we included another co-author (FS) as for review and editing input during the revision phase.

Thank you very much for your efforts and consideration.

Yours sincerely,

Prof. Dr. K. C. Huth, MME
